# Promoting physical activity and health in Hong Kong primary school children through a blended physical literacy intervention: protocol and baseline characteristics of the “Stand+Move” randomized controlled trial

**DOI:** 10.1186/s13063-021-05925-y

**Published:** 2021-12-20

**Authors:** Ming Hui Li, Cindy Hui Ping Sit, Stephen Heung Sang Wong, Yun Kwok Wing, Ching Kong Ng, Raymond Kim Wai Sum

**Affiliations:** 1 Department of Sports Science and Physical Education, The Chinese University of Hong Kong, Hong Kong SAR, China; 2 Department of Psychiatry, Faculty of Medicine, The Chinese University of Hong Kong, Hong Kong SAR, China; 3 Stewards Pooi Kei Primary School, Hong Kong SAR, China

**Keywords:** Physical literacy, Sit-stand desk, Physical activity, Children, Primary school, Sleep, Executive functions

## Abstract

**Background:**

Children predominantly remain sedentary in a traditional classroom. This study aimed to demonstrate the design and baseline characteristics of a three-armed intervention program that targeted enhancements in children’s physical literacy (PL), physical activity (PA), sleep, and executive functions by constructing an active classroom environment in primary schools. The blended approach involved organized PA participation during recess combined with changes to the classroom environment in response to the emphasis on nurturing children’s PL in Hong Kong.

**Methods:**

This blended “Stand + Move” intervention adopted a randomized controlled trial design to investigate its effectiveness in improving health-related aspects. Three groups were compared: (1) PA recess intervention, (2) blended (sit-stand desks and PA recess) experimental, and (3) control groups. In this 13-week intervention (from March to September 2019), 76 students (59.2% girls) were recruited and randomly assigned to the three groups. The primary outcomes were children’s PL and PA. The secondary outcomes were children’s sleep and executive functions.

**Results:**

Baseline data were evaluated. The mean age was 9.6 years [standard deviation = 0.61, range 9.0–12.0]. There were no significant differences between trial arms at baseline concerning any of the outcomes (all *P* = 0.06–0.89). Overall, 22.4% met the recommended PA guidelines, 36.8% met the sleep guidelines, and 10.5% met both guidelines. Three aspects of executive functions were evaluated: inhibition, executive control, and planning. Over half of the participants reported satisfaction with their perceived sleep quality.

**Conclusions:**

The designed intervention is regarded as an innovative strategy that incorporates sit-stand desks and PA breaks to reconstruct children’s traditional classroom environment. The baseline results suggest that intervention was satisfactory in reducing students’ sitting time and increasing their PA engagement. We demonstrated the benefits of this intervention on children’s PL, various sleep patterns, and executive functions. As expected, the designed intervention changes made to the classroom improved children’s health behaviors, as well as the support from stakeholders at schools and the children’s families. Our results also provided the desired evidence for policy reforms in teaching and learning strategies.

**Trial registration:**

ChiCTR ChiCTR2000035038. Registered on July 29, 2020—retrospectively registered

**Supplementary Information:**

The online version contains supplementary material available at 10.1186/s13063-021-05925-y.

## Background

Sedentary behavior (SB), defined as behavior requiring any low-level energy expenditure, such as sitting, lying, reclining, or expending < 1.5 metabolic equivalents [1], is an increasing issue in modern society. Prolonged sitting has been linked to poor physical health, as well as psychosocial and cognitive outcomes, irrespective of physical activity (PA) level [2]. According to the Hong Kong Report Card 2018, over 90% of Chinese school-aged children and youth insufficiently participate in PA [2]. The grade achieved for children’s SB was C-, indicating that over half (52%) of primary school children (mean age = 7.6 years) presented with < 2 h of screen time [2]. For children attending primary school, sitting is the predominant behavior in the classroom, except during physical education (PE) classes and breaks. Previous studies have shown that Hong Kong children spend up to 32.3% (approximately 4.9 h/day) of their waking time sitting [3]. Other studies have reported sitting times are up to 10 h/day [4]. High levels of SB have been negatively associated with cardiometabolic health risk markers, such as obesity and high blood pressure, cholesterol, and insulin levels in children [5, 6]. Importantly, SB can be tracked throughout childhood, adolescence, and adulthood [7]. Therefore, disrupting prolonged sitting habits and fostering healthy alternatives during childhood is paramount for promoting a healthy lifestyle in adulthood.

Positive associations have been found between PA and classroom behaviors and learning [8, 9]. Public health authorities have recommended that primary schools assist children in meeting the PA guideline of 60 min of moderate-to-vigorous PA (MVPA) through a comprehensive approach, incorporating activities during classes, recess, and before and after school [10]. The concept of physical literacy (PL) is also emphasized as an important ideology to encourage each child to move with “the motivation, confidence, physical competence, knowledge, and understanding to value and take responsibility for engagement in PAs for life” [11], which was first introduced to PE teachers in Hong Kong through a continuing professional development program provided by the Education Bureau [12].

### Fostering children’s PL and PA in the school setting

Cairney et al.’s conceptual framework suggests that the development of children’s PL early in life will influence their subsequent participation in PA and the related outcomes across the life course [13]. There is also evidence that among school-aged children, PL is the foundational component contributing to various health indicators, with PA playing a mediating role. Therefore, fostering PL and PA in school-aged children is of paramount importance. PA interventions in school-aged children must align with relevant systems (for example, health, education, local government, etc.) and have the scope to be feasible, cost-effective, and policy-relevant to achieve maximum impact and effectiveness [14]. Interventions focusing on the school environment could be a feasible, convenient, and cost-effective measures for developing children’s PL, encouraging PA, and reducing prolonged sitting time [15, 16]. To foster children’s habit of engaging in PA and reducing sedentary time, strategies that break up prolonged sitting and facilitate standing and moving seem feasibly beneficial for their health. Furthermore, children’s sitting behaviors tend to be habitual and without conscious thought. Their habitual behaviors may have developed from an early age during the school period. As such, favorable contexts or environments that motivate children’s participation in PA are essential targets for interventions [14]. For example, sitting is automatic and pervasive when classrooms are furnished with chairs and seated-height tables. Children are also under the control of their teachers/parents or other adults who are responsible for their behaviors and whose instructions may often require them to sit still in class or in front of the television. It is important to adopt novel strategies and assess effective ways to reduce SB in children.

In addition to reducing prolonged sitting in class, incorporating short bouts of activity throughout the school day could help children achieve the required amount of PA and foster their PL. For children to stay active, additional activities could be provided outside of break times and PE classes. Due to budgetary constraints and growing pressure on administrators and teachers to increase academic achievement scores, opportunities for PA are limited. An effective environmental approach to increase PA has been to incorporate activity breaks into primary school schedules [17]. PA breaks comprise low-cost, teacher-directed interventions that only require minimal support in the form of attractive resources, such as student posters, teacher notes, or music compact disks [18]. The intervention was reported to result in a significant increase in daily steps and children undertaking more PA during school hours than the controls [18]. The current study blended the “Stand + Move” design and also aimed to reduce prolonged sitting due to contextual constraints of a traditional classroom and increase PA engagement during school time.

### PA, SBs, and sleep

SBs, including high screen time, PA, and sleep, have recently been integrated as “movement behaviors.” To obtain optimal health, children are required to attain certain combinations of movement behaviors (for example, high PA/high sleep/low SB) [19]. These behaviors span the breadth of the movement continuum [20]. Recently, a holistic 24-Hour Movement Guideline for Children and Youth was proposed in response to global perspectives on children’s PL. The guideline emphasizes PA, SB, and sleep as three co-developmental movement behaviors related to a full range of movement within 24 h [21]. Specific daily recommendations are provided, including MVPA (≥ 60 min/day), screen time (≤ 2 h/day), and sleep (9–11 h/night for children aged 5–13 years and 8–10 h/night for adolescents aged 14–17 years). A PL-based comprehensive approach that incorporates PE, recess, and PA opportunities during both the classroom period and before and after school may assist children in meeting the movement guideline within a 24-h period [10]. To foster a lifelong active and healthy lifestyle, interventions should target a blended design to improve primary school children’s PA and PL and enable them to reduce prolonged sitting.

### PA interventions on executive functions (EFs) and sleep behavior

Previous studies have provided evidence that in early childhood, PA is positively associated with cardiometabolic, physical, and psychosocial development, as well as in improving children’s motor and cognitive development [22], especially EFs. It comprises a series of higher-order cognitive processes that develop rapidly during the early years [23] and routinely includes aspects of memory, inhibitory control, and cognitive flexibility to organize or coordinate behaviors when performing complex tasks [24]. Other studies have shown that sleep [25], PA [26], and screen-based SB [27] are associated with psychological health and early EFs; however, the combined influences of these behaviors are unclear. Few studies have investigated the integrated associations between movement behaviors and cognition in early childhood. To date, only one observational study has provided evidence that there are positive associations between cognition and each additional recommendation [28]. Given that movement behaviors are interrelated, health benefits may be optimized when all components are considered [20].

The aggregation of movement behaviors should also consider reducing prolonged sitting by focusing on decreasing typically observed SB among primary school children before their transition into adolescence. A study that explored a reduction in prolonged sitting found that it often contributed to decreased attention in class [29]. Previous literature reflects a lack of clarity in terms of the relationship between SB or PA and the different aspects of cognitive function. Therefore, it is necessary to include EFs as a measure to evaluate the effectiveness of active classroom intervention. These aspects of EF include not merely remembering important details and updating rules but also refer to inhibiting movements, adapting to different situations, and planning and acting in anticipation of an event, which together, lead to improved academic achievements [30].

Sleep has been associated with sedentary behavioral patterns, such as prolonged screen time. According to Must and Parisi [31], these two factors may operate in concert with one another, thereby increasing the likelihood of the child becoming obese. One meta-analysis showed that SB is associated with an increased risk of insomnia and sleep disturbance in adults aged 18+ years old [32]. A recent systematic review indicated that moderate PA seems to be more effective than vigorous activity in improving sleep quality [33]. However, research examining sleep quality and SB or PA has mainly focused on adults, elderly people, or patients. Hartescu, Morgan [34] found that walking exercises (≥ 150 min per week) were significantly associated with a reduced likelihood of insomnia symptoms, and walking levels significantly predicted the likelihood of sleep onset or sleep maintenance problems. Rogers, Courneya [35] conducted a PA intervention for breast cancer survivors and found that PA significantly improved the perceived sleep quality global score. Based on these findings, the effects of a blended PL intervention (increasing PA and reducing SB) on the sleep behavior of children should also be considered.

As school-aged children, during most lessons, are expected to sit in the traditional classroom setting, outside their limited PE lessons, it seems impossible for them to meet the movement guidelines within each 24-h period [36]. To reduce children’s sitting time and promote PA, practical strategies are needed to reconstruct the classroom environment so that children can develop PL and reduce their SB [37]. The “Stand + Move” program (combining sit-stand desks with PA breaks) is one strategy that can be feasibly achieved to develop PL in the school setting and reduce SB. Unlike traditional classroom desks, sit-stand desks are height adjustable, enabling the child to alternate between sitting and standing postures. A previous study reported a significant increase in SB in children aged 11 years and older relative to children of younger age groups [38]. Hence, it is necessary to reduce typically observed SB among primary school children before adolescence.

To the best of our knowledge, interventional studies targeting height-adjustable tables and PA breaks have not been conducted in a school setting. As such, this pioneering study incorporates sit-stand desks and PA breaks for reducing students’ sitting time and increasing their PA engagement. Moreover, the study aimed to investigate the effects of the intervention on children’s PL, sleep patterns, and executive functioning. The goal is ultimately to promote an all-around healthy lifestyle for students in Hong Kong primary schools. It was hypothesized that the blended “Stand + Move” group, compared with the single “Move” group and the control group, mostly showed an increase in (1) their self-perceived PL and actual PL; (2) objectively measured PA, SB, and sit-stand transitions; (3) EF in the domains of inhibition, executive control, and planning; and (4) sleep patterns, including sleep duration, sleep hygiene, sleep disturbance, and daytime sleepiness. Both the individual level (the self-reported and objectively measured outcomes) and the group level (comparing differences between groups) were included. In this report, we demonstrated the study protocol and baseline characteristics of the intervention program using a randomized controlled trial (RCT) design and reported the baseline characteristics of the trial population according to the CONSORT Statement for randomized-controlled trials (Additionall file 1) [39].

## Methods

### Study design

This study aimed to demonstrate the design and baseline characteristics of a three-armed intervention program that targeted enhancements in children’s PL, PA, sleep, and EFs by constructing an active classroom environment in primary schools. The “Stand + Move” intervention was designed as a three-arm RCT study to evaluate the effectiveness of the blended “Stand + Move” intervention program. Outcome data were collected at baseline, post-intervention, and 3-month follow-up via self-perceptions and objective measures of all variables. Baseline data were collected before the intervention in the two intervention arms. The post-intervention data were collected from all groups successively, 13 weeks after the completion of the intervention, to determine the intervention’s effect on children’s SB. Follow-up measurements in all groups were performed 3 months after the post-intervention measurement. Baseline data were collected between September and November of 2020. Measurements at post-intervention and follow-up were obtained during 2021.

**Fig. 1:**
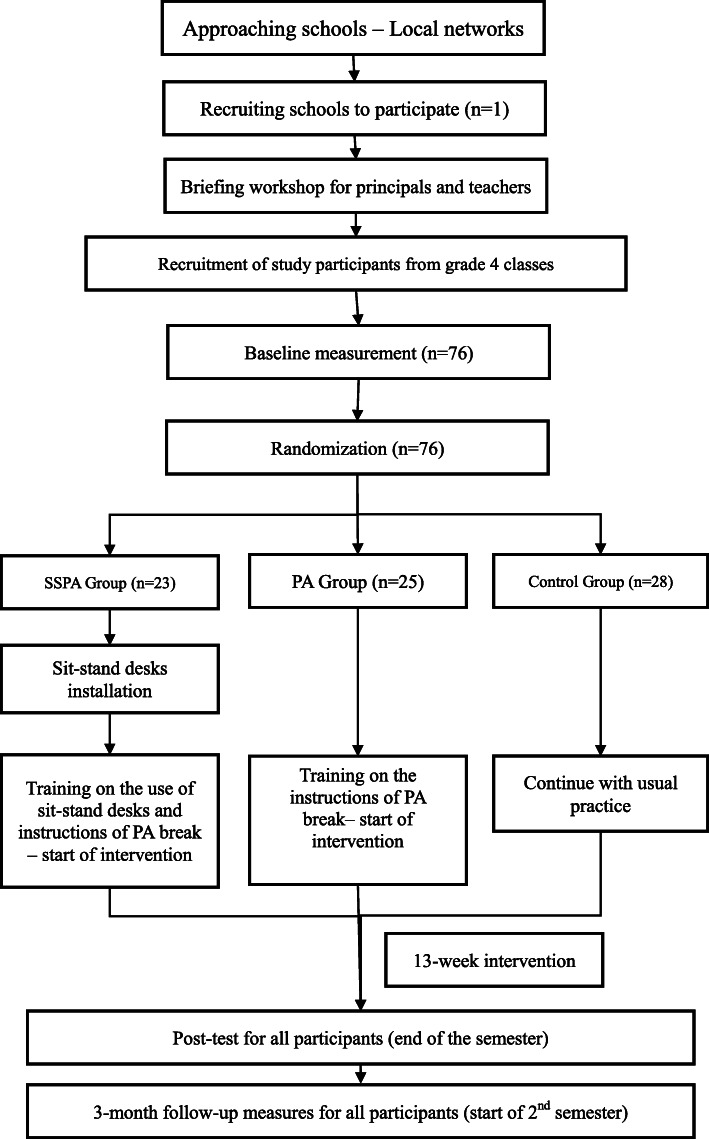
Flow diagram for the “Stand+ Move” intervention using CONSORT guidelines for RCT

The present study focused on 4th grade primary school students (9–10 years old) in Hong Kong. Ethical approval was obtained from the Survey and Behavioral Research Ethics of the Chinese University of Hong Kong. Following the initial recruitment process, baseline assessments were conducted at participating schools. Prior to their participation, written informed consent was provided by the parents or guardians of all children. All personal information about potential and enrolled participants were kept confidential before, during, and after the trial. Only the project members had access to the final trial dataset and only for data analysis. Figure 1 shows the study’s flow chart. The participants were randomly assigned (using Google Random Number Generator) into one of three groups: a single PA break intervention group (PA), a blended intervention group (combining sit-stand desks and PA breaks; SSPA), or a control group (remaining normal class schedule; CG). A blended intervention design was adopted, as it was considered an innovative design for disrupting prolonged sitting and improving engagement in PA, a pragmatic measure of school-aged children’s PL [40]. This intervention was regarded as a key facilitator of the Comprehensive School PA Program, a necessary approach for fostering school-aged children to develop their PL, considering that schools play a critical role in reshaping both social and physical environments, as well as providing information, tools, and practical strategies to help students adopt healthy lifestyles [10]. Primary outcomes included children’s PA level, including MVPA, sedentary time, and PL level (self-perceived and actual PL) [41]. Secondary outcomes consisted of children’s personal status, such as body weight and height, performance in EFs, academic achievement, and changes in sleep patterns and duration. As the current study was led by teachers who were required to implement the interventions in schools, they were blinded to group allocation. Students were not informed about the research aims and related information and were therefore blinded to group allocation. Research assistants who were responsible for data collection were also not blinded to the group allocation.

### Experimental intervention

Thirty sit-stand desks were placed with the standard desks in one 4th grade class of students in the intervention school for one semester. The research team included support teachers who facilitated class scheduling to ensure that the sit-stand desks were used by all children for the required amount of time. It is recommended that each student should use the sit-stand desks for at least 1 h/day across the week [42]. The research plan of reducing prolonged sitting every 15 min during two regular classes (each class before the recess) per day could ensure that all children in the “Stand + Move” group had access to the sit-stand desks for at least 1 h/day across the week [43]. Stools or chairs were available and children were free to choose whether to sit or stand outside the required period. A briefing workshop for school principals, teachers, and parents was conducted to introduce the program. Consent forms were distributed to all parents of grade 4 students training on the sit-stand desks. Stools or chairs remained in the classroom for students to freely use when they were at the sit-stand desks. For the two intervention groups, PA breaks of up to 15 min in duration, twice a day across the week, which included games, such as skipping rope, shuttlecock kicking, and hide-and-seek in a specific area supplemented with several minutes of cooling down were scheduled.

### Control arm

To compare the effects of the intervention against the usual practice (that is, the provision of standard classroom desks), students in the control arm were requested to continue their usual practice and lesson delivery, and no environmental changes were made to their classrooms. The same study measurements as those in the intervention schools were collected at the same time points from the students in the control group.

### Participants and recruitment

As previous research reported a significant increase in SB in children aged 11 years and older [38] and to reduce the typically observed SB before transitioning into adolescence, this study recruited students from the 4th grade in Hong Kong primary schools (students aged 9–10 years). G-power software was used to calculate the sample size with an alpha of 0.05, power of 80%, and effect size (*r*) of 0.3 [44]. Since a full desk allocation system (a sit-stand desk for every child) guaranteed optimal health benefits for the children as they could have maximum exposure to the desks, only one school was approached and recruited. Although a non-probability convenience sampling was adopted, a total of 76 participants were randomly assigned to one of the three conditions in a 1:1:1 ratio (Fig. 1). Children were excluded if they (a) were not in 4th grade, (b) had a disability that prevented periods of standing, or (c) had an injury or illness that limited performing normal daily tasks.

Consent forms were distributed to all the parents of all 4th grade students by the school teachers. A briefing session covering the aims and procedures of the intervention was held for all teachers and parents who agreed to participate. Participants’ personal data were kept confidential. Participants were informed that they could voluntarily withdraw from the project at any time, without prejudice. Upon reaching an agreement with the principals, teachers, and parents and after obtaining informed consent, the trained appraisers started data collection.

## Baseline measures

### Primary outcomes

#### PL—actual level

The body mass index (BMI, kg/m^2^) was calculated from the measured weight (nearest 0.1 kg) and height (nearest 0.1 cm). PL was assessed using the Chinese version of the Canadian Assessment of PL, second edition (CAPL-2, Chinese) [45], a comprehensive protocol that can accurately and reliably assess a broad spectrum of skills and abilities that contribute to and characterize the PL level of a participating child [46]. It is comprised of four domains: daily behavior, physical competence, knowledge and understanding, and motivation and confidence. The total achievable score for this assessment was 100. Daily behaviors were addressed in two parts: objectively measured step counts and self-reported MVPA (that is, the number of days in a week that children engaged in activities that made them breathe hard and their heart beat fast). The total achievable score for this assessment was 30 points. Physical competence consisted of three parts: (i) FitnessGram 15 m/20 m Progressive Aerobic Cardiovascular Endurance Run (PACER) [47] to evaluate aerobic fitness; (ii) Plank Assessment of Torso Strength [48] for testing musculoskeletal endurance related to back health, the ability to stabilize the body, and the functioning of both the upper and lower limbs; and (iii) the Canadian Agility and Movement Skill Assessment (CAMSA) for assessing motor competence [49]. The total achievable score for this assessment was 30 points. The knowledge and understanding domain assessed a child’s PL-related knowledge, with five questions equaling 10 points. The motivation and confidence domain evaluated a child’s confidence in their ability to be physically active, as well as their motivation to participate in PA. A revised version of the “What’s most like me,” the Children’s Self-perceptions of Adequacy in and Predilection for Physical Activity questionnaire [50], was adopted to assess this domain with a total of 30 points, which was adequately evaluated for a model of fit after revisiting the PL concept [51]. The whole CAPL-2 (Chinese) model was reported to have good construct validity: chi-square (*χ*^2^ = 70.16, *df* = 43, *p* < 0.05), root mean square error of approximation (RMSEA) = 0.04, 90% confidence interval (CI; 0.024–0.062), CFI = 0.94, TLI = 0.90, for the evaluation of children’s PL.

#### Perceived PL

PL perceptions were assessed using the adolescent version of the Perceived Physical Literacy Instrument, a nine-item questionnaire [52]. Each response was rated on a 5-point Likert scale ranging from strongly disagree to strongly agree. Adapted from a previous version constructed by PE teachers [53], the validity of the current questionnaire was confirmed through a confirmatory factor analysis: chi-square (*χ*^2^ = 321.54, *df* = 24, *p* < 0.05), CFI = 0.95, RMSEA = 0.08, and standardized root mean square residual = 0.04. Furthermore, the questionnaire showed acceptable reliability, with *α* values ranging from 0.68 to 0.76.

#### PA

Children’s PA was monitored using two different types of activity devices, namely, the ActiGraph GT3X+ [54] and activPAL™ accelerometers (PAL Technologies, Glasgow, UK). The ActiGraph monitors were worn on the children’s waists for 7 consecutive days. Data were collected in 15 s epochs to account for children’s natural activity levels, which generally occurred in short bouts [55] as they were shown to present the most acceptable classification accuracy for accelerometer use among children. Evenson cut-points (MVPA ≥ 2296 counts min−1) were applied to the intensity levels. The ActiGraph monitors had to be worn for at least 8 h/day for a minimum of 4 days, with at least one valid weekend included [56]. The accelerometers could be removed only during water activities, such as showering or swimming, and the participants had to provide details in their log sheets. The activPAL monitors were worn on the midline of children’s right thighs and could be used to detect limb positions, such as sitting/lying, standing, and stepping [57]. Similar to the protocol used for the ActiGraph, a continuous 7-day-wearing waterproof protocol was adhered to, to ensure the monitoring of children’s PA and SB for the entire 24 h. The activPAL data were divided into 15-s periods, meeting a minimum requirement of 3 valid weekdays and 1 valid weekend day [58, 59]. The activPAL data were summarized as the time spent sitting/lying, standing, and stepping.

### Secondary outcomes

#### EFs

##### Inhibitory control

EFs, including inhibition, executive control, and planning, were assessed by three computer-based tasks, all of which were performed using the Inquisit 5 platform. Participants were required to perform three tasks, one by one, in a quiet room under the supervision of an instructor who was trained prior to testing. Inhibition control was examined using a modified version of the Eriksen flanker task [60]. This task consisted of five arrows on a screen, and participants were asked to determine the direction of the target arrow in the middle. The arrows pointing to the left “<” and right “>” directions required a right and left keyboard button response, respectively. The two flanker arrows on each side of the target arrow worked as distractors and appeared as either congruent trials >> >> > “>>>>>” or congruent trial “>><>>.” Each stimulus was shown for 120 ms, and the participants were required to respond within 200 to 1750 ms from the onset of the arrows, for a valid response. This task contained 4 practice trials and 20 test trials, with an equal number of congruent and incongruent trials occurring in a random order. The outcomes included two domains: accuracy (percentage of correct responses) and reaction time (number of ms for correct responses).

##### Executive control

Executive control was measured using the classical version of the Wisconsin Card Sorting Test with a standard number of 128 cards [61]. This task consisted of four key cards and 128 response cards. Participants were instructed to sort the response cards, shown at the bottom of the screen, according to the characteristics of the key cards presented on the screen’s upper side, comprising the following categories: colors (red, green, yellow, and blue), forms (triangles, stars, crosses, and circles), and numbers [1–4]. The instructor was permitted to provide instructions relating to the categories either prior to or during the task, while feedback on “correct” or “incorrect” was presented after each selection. Each participant took approximately 20 min to complete the task. Both total and perseverative errors were recorded as executive control variables, since an increase in any of these variables suggested executive control impairment [62]. While the calculation of total errors was based on the number of times participants matched a card incorrectly, perseverative errors were based on the participants’ continuing to follow the previous error rule.

##### Executive planning

The Tower of London Task, a widely administered neuropsychological assessment, was used to measure the planning aspects of EFs [63]. The task consisted of a practice trial, and 12 test trials required participants to move beans to solve problems. A graph on the screen showing three vertical pegs with graded heights and each holding beans (either 3, 2, or 1) were presented to the participants, who had to move the beans so as to be identical to the goal graph, without violating the rules [64]. Each participant took approximately 20 min to complete the task. Both the total correct and total move scores were derived for the analysis, given that these variables were found to be influenced by aerobic and resistance exercises [64].

#### Sleep patterns

The Children’s Report of Sleep Patterns questionnaire, containing 60 items pertaining to three modules (sleep patterns, sleep hygiene index, and sleep disturbances scale) was used to measure different aspects of sleep among children aged 8 to 12 years [65]. The questionnaire’s psychometric properties were tested in a population of 456 children using a multi-method, multi-reporter approach and were reported to have good reliability and validity. This assessment was performed in this study. Considering the negative impact of sleep loss on grades and overall daytime functioning in children, daytime sleepiness was also assessed using the Pediatric Daytime Sleepiness Scale (PDSS) [66]. This was a parent-reported instrument consisting of eight items with > 0.40 acceptable factor loadings. The internal consistency of the total 8-item scale (factor 1, PDSS) was *α* = 0.81/0.80 for the split-half samples.

### Data collection procedures

During the first stage of data collection, participants were required to complete both the CAMSA and Plank Assessment of Torso Strength during the PE class. The participants were divided into two groups, with one or two appraisers per group, and rotated around the stations (one test per station) until the assessment was completed. Prior to the CAMSA, they watched two test presentations performed by an appraiser. During the first presentation, the appraiser covered the entire course at a slow pace with detailed verbal descriptions of each skill. The second presentation was conducted at full speed, with the appraiser ensuring that skill accuracy was maintained. The participants were required to practice twice at full speed while maintaining their skill accuracy. Each participant’s highest combined time and skill score was recorded as the final grade. In the Plank test, the participants first watched the demonstrations. Thereafter, stopwatches were used to record the time point at which each participant achieved the correct posture. There was a warning when the participant’s position was too low or high or if the posture was not maintained. The recording was stopped when the participants shifted their positions a second time.

During the second stage of data collection, participants’ aerobic fitness was monitored based on their participation in the PACER 15 m/20 m shuttle run during their scheduled PE classes. Due to limited space, all participants ran from one marker to another, set 15 m apart, while keeping pace with a prerecorded Cantonese cadence. The total number of laps achieved by each participant was recorded and subsequently converted to the standardized 20 m PACER score using the FitnessGram PACER Conversion Chart [47]. Finally, ActiGraph and activPAL accelerometers were distributed to the participants to monitor their step counts, PA, and SB for 7 consecutive days.

Height and weight measurements, questionnaire completion, and participation in the cognitive tests all occurred during music, science, or other classes, and not during the PE classes. Two participants visited the quiet experimental room at a time to complete the aforementioned measurements under the instruction of two trained helpers.

### Data analysis

Descriptive statistics are expressed as frequencies, ratios, and means with standard deviations. The Shapiro–Wilk and Levene’s tests were used to check the normality and homogeneity of the data. A multivariate analysis of variance test was used to assess between-group comparisons at baseline. Prior to analyzing the data, several methods were adopted to replace any missing values in the outcome variables. The missing values in the physical competence and daily behavior domains of PL were calculated according to the fraction provided in the CAPL-2 (Chinese) manual. A score could still be calculated when a maximum of one protocol was completely missed [51]. The 15 missing raw scores in the physical competence domain were replaced using the recommended algorithm within the CAPL-2 (Chinese). For measuring PA, an individual information-centered approach was adopted to substitute missing data points [67]. This has been demonstrated as an effective method and superior to the group information-centered methods for handling missing accelerometer data when data were collected for 7 days.

It is expected that when the immediate post-test and 3-month follow-up tests are completed, a two-factor mixed-design analysis of covariance will be conducted to assess the change in dependent variables over the three time points between groups, separately. Adjustments were made for sex, age, and BMI.

### Process evaluation

Process evaluation (often called *formative evaluation*) aims to improve a policy or program as it is being implemented [68]. Regarding feasibility, evaluation should check whether and to what degree the implementation is accomplished, such as context, reach, fidelity, acceptability implementation, impact, acceptability, and sustainability over time through a pragmatic design [69]. In the current study, a qualitative methodology was adopted to examine the perceptions and experiences of key stakeholders in the intervention schools, as the interpretivist paradigm illustrates that human action and interaction in the school setting is subjectively evaluated through individual meaning-making [70]. Key stakeholders, such as teachers and students, are inherently associated with the effectiveness of the blended “Stand + Move” intervention. This method included semi-structured interviews with the teachers involved in the intervention group and focused on groups with randomly selected students after measurements to avoid its influence on the results. Moreover, classroom observations were conducted during the intervention period by research team members, who recorded field notes based on these observations [71], including positive or negative responses to the enforced sit-stand desks utilized during class, the children’s attitude towards sit-stand desks during the class and PA breaks during the recess, and sitting and standing behavior immediately after the enforced sit-stand desk implementation.

### Baseline results

 A total of 76 students (59.2% girls, *M*_age_ = 9.6 years [standard deviation = 0.61, range 9.0–12.0]) were evaluated after addressing the missing data. Daytime sleepiness data were not available for eight participants (SSPA = 3; PA = 1; and CG = 4) as the questionnaires were not returned from their parents. Table 1 shows the baseline demographic characteristics of all the participants. The majority (69.4%) of the participants’ parents obtained at least college or university level of education, and the family house type and income status reflected the socioeconomic demographic status of the participants in the three groups. Table 2 displays the baseline descriptive statistics of the primary outcomes, with group differences. When the activPAL monitors were worn, nearly half of the participants reported allergic symptoms relating to the waterproof hypoallergenic tape (3M Tegaderm™; 3 M Health Care™, St. Paul, MN; 10 cm × 10 cm), leaving only 41 valid data for the analysis (SSPA = 22; PA = 7; and CG = 11). There was no significant difference between the groups regarding any of the primary outcomes (*P* = 0.055–0.808). The only exception was a significant difference observed in the standing time measured by the activPAL between the PA and CG groups (*p* = 0.017). The baseline descriptive statistics of the secondary outcomes with group differences are shown in Table 3. No significant group differences were found in any of the secondary outcomes (*P* = 0.133–0.886). Several aspects of sleep are shown in Table 4. These aspects reflected the children’s perceived sleep quality and habits. Over half of the participants in each group reported satisfactory sleep patterns in terms of duration (SSPA = 60.9%; PA = 80%; and CG = 75%). Very few children reported their perceived sleep quality as poor (SSPA = 4.3%; PA = 4.0%; and CG = 3.6%). According to the 24-Hour Movement Guidelines in Children and Youth [72], 22.4% of the participants met the recommended PA guidelines, 36.8% met the sleep guidelines, and 10.5% met both guidelines.

**Table 1:** Baseline demographic characteristics of the study participants

	SSPA (***n*** = 23)	PA (***n*** = 25)	CG (***n*** = 28)
**Demographic**			
Gender (female)	15 (60%)	16 (57.1%)	17 (60.7%)
Age, mean (SD)	9.7 (0.7)	9.6 (0.6)	9.6 (0.6)
Height, mean (SD)	136.3 (6.6)	134.8 (8.7)	136.3 (4.3)
Weight, mean (SD)	31.6 (7.5)	32.3 (9.0)	31.6 (6.3)
BMI (kg/m2), mean (SD)	16.8 (3.0)	17.3 (3.1)	16.9 (2.8)
**Socio-demographic characteristics**			
Parent’s highest level of education completed			
Secondary school (%)	31.8	41.7	19.2
College (%)	31.8	16.7	26.9
University (%)	34.8	33.3	50.0
Postgraduate (%)	0	8.3	3.8
House type			
Public housing estates (%)	21.1	0	26.3
Private housing estates (%)	57.9	89.5	68.4
Single House (%)	5.3	10.5	0
Others (%)	15.8	0	5.3
Family monthly income			
Less than 40,000 (%)	40.9	21.7	29.2
From 40,000 to 70,000 (%)	36.4	47.9	45.8
Greater than 70,000 (%)	22.7	30.4	25

*Abbreviations*: *BMI* body mass index, *SSPA* sit-stand desks and PA blended group, *PA* single PA group, *CG* control group

**Table 2:** Baseline characteristics of primary outcomes and group comparison

	SSPA (***n*** = 23)	PA (***n*** = 25)	CG (***n*** = 28)	***p*** =
*Physical literacy—actual level*				
Motivation and confidence	22.8 ± 4.8	21.4 ± 5.0	22.0 ± 4.8	0.619
Physical competence	14.7 ± 5.7	13.3 ± 3.5	14.6 ± 4.0	0.351
Knowledge and understanding	4.5 ± 1.9	4.6 ± 1.3	4.0 ± 1.8	0.254
Daily behavior	10.5 ± 3.5	9.4 ± 3.2	10.8 ± 3.6	0.330
*Perceived physical literacy*				
SS_PPL	11.3 ± 2.5	10.9 ± 1.9	11.0 ± 1.6	0.760
SE_PPL	11.4 ± 2.4	11.1 ± 1.4	11.1 ± 1.9	0.808
KU_PPL	12.6 ± 2.5	12.1 ± 1.6	12.5 ± 1.5	0.658
*Physical activity—ActiGraph*				
Total wear time (min)	4680.3 ± 1412.2	4387.8 ± 1672.0	3844.7 ± 1678.3	0.191
Sedentary (%)	67.3 ± 7.4	66.8 ± 5.1	68.3 ± 7.2	0.661
LPA (%)	28.0 ± 5.2	28.0 ± 4.2	26.8 ± 5.8	0.667
MVPA (%)	5.1 ± 2.0	5.2 ± 1.8	5.0 ± 2.0	0.622
*Physical activity—activPAL*				
Sitting time^a^	1183.1 ± 81.0	1212.6 ± 82.2	1184.3 ± 85.1	0.700
Standing time^a^	220.8 ± 44.4	190.5 ± 49.5	244.7 ± 42.7	0.055^#^
Stepping time^a^	125.7 ± 21.3	105.8 ± 28.2	114.0 ± 23.3	0.111
Sit-to-stand transitions^a^	107.8 ± 21.0	98.3 ± 31.5	103.1 ± 20.1	0.612

^#^*p* < 0.10

^a^*n* = 41

*Abbreviations*: SS_PPL, SE_PPL, and KU_PPL are the attributes of perceived physical literacy; *LPA* light physical activity, *MVPA* moderate-to-vigorous physical activity, *SSPA* sit-stand desks and PA blended group, *PA* single PA group, *CG* control group

**Table 3:** Baseline characteristics of secondary outcomes and group comparison (M, SD)

	SSPA (***n*** = 23)	PA (***n*** = 25)	CG (***n*** = 28)	***p*** =
*Sleep*				
Sleep time (h)	8.2 ± 0.7	8.3 ± 1.2	8.4 ± 0.8	0.498
Sleep hygiene	32.6 ± 5.1	31.0 ± 7.6	32.5 ± 7.8	0.680
Sleep disturbance	27.6 ± 5.8	26.0 ± 7.8	28.0 ± 10.8	0.667
Daytime sleepiness^a^	13.6 ± 5.6	14.6 ± 5.0	13.5 ± 5.8	0.786
*Inhibition*				
Accuracy—congruent (%)	99.6 ± 2.0	97.8 ± 4.2	98.6 ± 4.5	0.355
Accuracy—incongruent (%)	94.2 ± 18.6	90.0 ± 21.3	95.0 ± 12.0	0.580
RT—congruent (ms)	626.6 ± 167.4	698.8 ± 203.7	642.2 ± 174.1	0.256
RT—incongruent (ms)	795.6 ± 353.2	774.7 ± 338.6	732.7 ± 239.0	0.769
*Executive control*				
Total errors^	43.3 ± 19.2	51.4 ± 15.2	52.9 ± 18.0	0.138
Perseverative errors^	7.5 ± 2.4	6.9 ± 4.4	8.3 ± 7.2	0.591
*Planning*				
Total correct	74.0 ± 12.7	73.3 ± 11.4	71.6 ± 14.4	0.886
Total move scores	29.0 ± 3.2	29.4 ± 2.5	27.8 ± 3.8	0.133

^a^*n* = 68

^Lower scores indicate better performances

*Abbreviations*: *RT* reaction time, *SSPA* sit-stand desks and PA blended group, *PA* single PA group, *CG* control group

**Table 4:** Baseline characteristics of sleep habits among the study participants

	SSPA (***n*** = 23)	PA (***n*** = 25)	CG(***n*** = 28)
**Sleep habits**			
Usual night wakeups			
5–7 times/week (%)	8.7	8.0	7.1
1–4 times/week (%)	17.4	16.0	10.7
2 3 times/month (%)	43.5	28.0	28.6
Never (%)	30.4	48.0	53.6
Back to sleep latency after wakeups			
Never wake up (%)	36.4	44.0	42.9
Very quickly (%)	18.2	12.0	14.3
5–10 min (%)	31.8	32.0	25.0
10–30 min (%)	4.5	8.0	14.3
More than 30 min (%)	9.1	4.0	3.6
Nap frequency			
Never (%)	39.1	36.0	35.7
Never unless sick (%)	47.8	36.0	39.3
Sometimes (%)	13.0	24.0	17.9
Almost every day (%)	0	4.0	7.1
Perceived sleep duration			
Too much (%)	13.0	4.0	3.6
Right amount (%)	60.9	80.0	75.0
Too little (%)	26.1	16.0	21.4
Perceived sleep quality			
Great (%)	13.0	20.0	35.7
Good (%)	56.5	48.0	46.4
Okay (%)	26.1	28.0	14.3
Poor (%)	4.3	4.0	3.6

*Abbreviations*: *SSPA* sit-stand desks and PA blended group, *PA* single PA group, *CG* control group

## Discussion

The current study pioneered the incorporation of sit-stand desks and PA breaks as an active strategy to not only reduce students’ sitting time and increase their PA engagement but also investigate the benefits of this intervention on children’s PL, various sleep patterns, and EFs. This was a multi-level appraisal to assist students in pursuing a healthy lifestyle among Hong Kong primary schools. As children spend the majority of their waking time being sedentary at school, environmental changes in the classroom could be an effective way of reducing their sitting time [10]. Although sit-stand desk interventions have been employed in many Western countries to promote children’s health, previous studies were restricted to only reducing or disrupting prolonged sitting time. Thus, providing an active environment for children, such as combining PA classroom breaks with sit-stand desks, would be a good approach for increasing their engagement in PA, especially MVPA, as public health authorities have recommended that primary schools are responsible for helping children meet their 60 min/day PA goal [73].

The baseline characteristics showed this intervention was satisfactory, with no significant difference between the groups for any of the variables. Thus, the current study was continued smoothly through the implementation of the intervention, immediate post-test, and 3-month follow-up tests. As this study adopted the incorporation of sit-stand desks and PA breaks into the blended “Stand + Move” intervention, it optimized a blended approach to increase children’s engagement in PA and PL development. As expected, the current intervention provided evidence for the enhancement of children’s PL, a co-development of PA, SB, and sleep, and executive functioning within a 24-h period for Hong Kong primary school students.

Although traditional classrooms and educational institutions in Hong Kong encourage the adoption of innovative teaching methods, such as incorporating fundamental movement skills into the PE class content, this is the first attempt at a research-based intervention combining children’s learning environment with healthy lifestyle nurturing. Many countries that rank within the top 25 for obesity prevalence have emphasized the importance of the PL concept as a guiding ideology in their policies and programs in response to both global PA decline and SB accumulation [74]. With its focus on encompassing “motivation, confidence, physical competence, and knowledge and understanding,” to value and take responsibility for engagement in PA for life [75], PL would work as a guiding ideology for conceptualizing the current research and providing scope for embodied enrichment on the pathway toward an active lifestyle.

This experimental research served as a pioneering study, combining sit-stand desks with PA active breaks, in Hong Kong’s primary school environment. The RCT aimed to investigate the effects of a classroom-based teacher-led intervention on children’s PL, PA, sleep, and EFs. The present study is significant and innovative in that it reconstructed an active classroom environment for students in primary schools to promote a healthy lifestyle, physical fitness and motor skills, and EFs, and enhances the healthy pattern of sleep through comprehensive objective and subjective measures. The 24-Hour Movement Guideline for Children and Youth emphasizes PA, SB, and sleep as three co-developmental movement behaviors related to the full scope of movement within a 24-h period [21]. To the best of our knowledge, no previous investigations have combined PL, PA, and sleep changes using environmentally driven strategies, while simultaneously including comprehensive measures within different fields. The current study also outlined the evidence-based, health-related benefits of the “Stand + Move” intervention for expanded use with Hong Kong primary school-aged children. It supports the use of sit-stand desks and active classroom strategies for primary schools. Furthermore, the study emphasized the PL concept, which has been adopted as the new standard to evaluate PE outcomes in many other countries. This study suggests a possible longitudinal benefit of incorporating sit-stand desks in improving students’ EFs and academic achievements.

However, the baseline results of this study have some limitations. First, as a full desk allocation system (a sit-stand desk for every child) was necessary to guarantee optimal health benefits for the participants, only one school was recruited for this study. Therefore, the present intervention should be interpreted with caution regarding the results’ generalizability. Second, when adopting the activPAL for use in school-aged children, a high drop-out rate was observed. Although several strategies were applied to avoid unnecessary removal of the activPAL, such as using alcohol pads to clean the skin before attachment and placing cartoon stickers to motivate the children [76]. This was consistent with previous studies conducted on preschool children [77] and primary school-aged students [76] because skin irritations, such as itchy skin and allergic reactions were prevalent in a moist environment, such as in Hong Kong with its humid weather. Data collected via the activPAL in this study may be insufficient.

Due to the high prevalence and harmful effects of sedentary lifestyles in children and youth, effective interventions to motivate participation in PA in different contexts are warranted. Considering this study’s valuable and detailed research design, we hypothesized that its “Stand + Move” RCT would be effective in increasing several aspects of children’s PL, PA, sleep, and EFs. The study’s direct beneficiaries were the participants and the stakeholders: (1) children in Hong Kong primary schools participating in the “Stand + Move” intervention. They enhanced their PL and co-developed their PA, SB, and sleep, as well as their EFs and academic performance. The participants also benefited from receiving a report from their physical and psychosocial assessments. Although children in the control group were not involved in the sit-stand desk or PA break interventions, this intervention enhanced their understanding of their physical and psychological performance, both short- and long-term. (2) All stakeholders benefited as they had access to resources and relevant information related to the “Stand + Move” intervention, as well as the underlying rationales. Increasing participation in PA and reducing time spent in SB may inspire an active lifestyle in their PL journey. As a result of participating in this project, the stakeholders—principals, teachers, parents, and guardians—may have also benefited through enhancements in their children’s health-related physical and psychological development. Furthermore, the collaborative project enhanced awareness among organizations in Hong Kong, such as Non-Profit Organizations, Hong Kong Physical Fitness Association, etc., to promote such feasible and innovative classroom environments for pursuing healthy living through effective engagement, for both short- and long-term. (3) Policymakers in government departments, such as Hong Kong’s Education Bureau, Education Commission, and Quality Education Department benefited from the research results, which provided evidence to support their policy-related decisions in relation to reducing and disrupting SB, thus promoting PA throughout a child’s life. (4) The Hong Kong Physical Fitness Association benefited from providing professional training to PE practitioners in the evaluation of PL in children. A certificate named “Childhood Physical Literacy Leader Certificate” will be promoted among PE practitioners in Hong Kong to enhance their competence in the field of education.

## Conclusions

The current study pioneered the incorporation of sit-stand desks and PA breaks as an active strategy for reducing students’ sitting time and increasing their PA engagement. The study also investigated the effects of this intervention on children’s PL, various sleep patterns, and EFs. The baseline results suggest that this study was well designed and implemented and has been satisfactory so far. This blended classroom-based intervention is expected to provide empirical evidence of enhancements in school-aged children’s PL, PA, EFs, and sleep, with a special focus on meeting the movement guidelines within a 24-h period. Future studies are needed to provide a multi-level appraisal to assist Hong Kong primary school-aged students in pursuing a healthy lifestyle.

## Supplementary Information

**Additional file 1.** CONSORT 2010 checklist of information to include when reporting a randomised trial

## Data Availability

Due to data protection regulations, datasets cannot be made available to third parties.

## Abbreviations

BMI: Body mass index
CI: Confidence interval
CAMSA: The Canadian agility and movement skill assessment
CAPL-2, Chinese: Chinese version of the Canadian Assessment of PL, second edition
EF: Executive function
MVPA: Moderate-to-vigorous physical activity
PA: Physical activity
PACER: Progressive Aerobic Cardiovascular Endurance Run
PDSS: Pediatric Daytime Sleepiness Scale
PE: Physical education
PL: Physical literacy
RCT: Randomized controlled trial
RMSEA: Root mean square error of approximation
SB: Sedentary behavior
SSPA: Group receiving a combination of sit-stand desks and PA breaks
